# Epidemiology of Underweight among Infants in Rural Burkina Faso

**DOI:** 10.4269/ajtmh.21-0838

**Published:** 2021-10-25

**Authors:** Ali Sié, Mamadou Ouattara, Mamadou Bountogo, Clarisse Dah, Guillaume Compaore, Elodie Lebas, Jessica M. Brogdon, Ying Lin, William W. Godwin, Kieran S. O’Brien, Benjamin F. Arnold, Thomas M. Lietman, Catherine E. Oldenburg, for the Étude CHAT Study Group

**Affiliations:** ^1^ Centre de Recherche en Santé de Nouna, Burkina Faso;; ^2^ Francis I Proctor Foundation, University of California, San Francisco, California;; ^3^ Division of Epidemiology and Biostatistics, University of California, Berkeley, California;; ^4^ Department of Ophthalmology, University of California, San Francisco, California;; ^5^ Department of Epidemiology and Biostatistics, University of California, San Francisco, California

## Abstract

Infant undernutrition is thought to contribute to growth failure and mortality. We evaluated the patterns in underweight in a population-based sample of children aged 1–11 months in rural northwestern Burkina Faso. Data were collected during the baseline assessment of a community-randomized trial evaluating mass azithromycin distribution in Nouna District, Burkina Faso. A door-to-door census was undertaken for all households in all communities. Infants aged 1–11 months were weighed for weight-based dosing in the trial and their weights were used to calculate weight-for-age Z-scores (WAZ). Underweight was defined as WAZ ≤ 2. We evaluated the age patterns in WAZ and underweight by demographic, seasonal, and geographic characteristics. Of 7,109 infants, 6,077 had accurate weight and global positioning system (GPS) coordinate data (85.5%). Infants were a median of 6 months old (interquartile range [IQR] 3–8) and 48.4% were female. Mean WAZ was −0.68 (SD 1.6) and 19.0% were underweight. The WAZ decreased with increasing age, and the prevalence of underweight increased from 2.5% among 1-month-olds to 27.6% among 11-month-olds. Underweight was more common among boys than girls (22.1% among boys versus 15.6% among girls). Improved latrine use by the household was associated with increased WAZ, and this effect was stronger in male compared with female infants. Given the large burden of underweight among infants, interventions addressing undernutrition should specifically include infants.

## INTRODUCTION

The first 1,000 days of life (from conception through age 2 years) is considered an essential period for access to adequate nutrition to reduce the risk of adverse developmental outcomes.^1^^,^^2^ Growth failure occurring during this period can affect growth trajectories and cognitive development much later into childhood and adolescence.^3^ Some evidence has suggested that although undernourished infants can experience some catch-up growth, they remain behind their well-nourished peers in their growth trajectory.^4^^,^^5^ Weight-for-age Z-score (WAZ), which is a measure that combines both acute and chronic malnutrition, is predictive of mortality in children 6- to 59-month-olds.^6^^,^^7^ Children classified as underweight (WAZ <−2) represent a vulnerable subpopulation as they are at increased risk of morbidity and mortality compared with their well-nourished peers, and thus may benefit from targeted interventions. These interventions may be particularly valuable early in life, as early growth failure is predictive of worse outcomes later in childhood.^8^^,^^9^

The Central Sahel (Mali, Niger, and Burkina Faso) in West Africa is a particularly vulnerable region for child health and nutrition because of seasonal food insecurity, ongoing political instability, and climate change, which may alter and shorten growing seasons.^10^^,^^11^ The COVID-19 pandemic increases the risk of poor nutritional outcomes for children, especially in already vulnerable settings.^12^ In Burkina Faso specifically, undernutrition is common among children under 5 years of age, with approximately 25% of children classified as underweight in recent Demographic and Health Surveys.^13^ The infant and young child nutrition (IYCN) initiative in the Sahel aimed to achieved optimal feeding of >80% of children aged 0–24 months, with the ultimate goal of improving nutritional status, reducing mortality, and improving developmental outcomes for these children.^11^ This initiative recognized the Sahel as a critical region with continued high prevalence of underweight and high mortality rates, and identified lack of progress toward Millennium Development Goals related to reducing underweight in children in Burkina Faso.^14^ Given the paucity of data for children under 6 months of age in particular^15^ and the importance of undernutrition during infancy (<12 months of age) for predicting worse outcomes as children get older, data on underweight in infants from community-based surveys in the Sahel are necessary to gauge progress and identify gaps in achieving goals from the IYCN initiative. Here, we describe the epidemiology of underweight in infants aged 1–11 months in a population-based sample in rural Burkina Faso and evaluate potential determinants of undernutrition.

## METHODS

### Study setting.

This study took place as part of the baseline assessment for the Community Health with Azithromycin Trial (CHAT), a community-randomized trial of mass azithromycin distribution compared with placebo for the prevention of under-5 mortality.^16^ All measurements were collected before the administration of azithromycin or placebo. The CHAT study takes place in Nouna District in northwestern Burkina Faso and encompasses both the Nouna Health and Demographic Surveillance (HDSS) site^17^ and the area in the district outside of the HDSS. The HDSS covers approximately one-third of the district and has been in operation since 1992. The area is rural and agrarian. The lean season follows the rainy season, which lasts approximately from May through September. The annual harvest of cereal crops begins after the rainy season, in October.^18^^,^^19^ Healthcare for children under 5 years of age is provided for free through a network of *Centers de Santé et de Promotion Sociale*, which are nurse-led primary healthcare facilities that provide basic prevention and treatment services.^20^ Each primary healthcare facility serves a set catchment area, which typically includes several villages.

### Ethics.

This study was conducted according to the guidelines laid down in the Declaration of Helsinki^21^ and all procedures involving research study participants were reviewed and approved by the institutional review board at the University of California, San Francisco, and the Comité d’Ethique pour la Recherche en Santé (National Ethics Committee of Burkina Faso) in Ouagadougou, Burkina Faso. Written informed consent was obtained from the head of household for the household’s participation in the census and household survey, and from each child’s caregiver for their participation in the trial. The minimum age of assent in Burkina Faso is 12 years, and thus assent was not obtained for children in this study.

### Participants.

A door-to-door enumerative census was undertaken in each study community from August 2019 through February 2020. Members of the study team visited each household in every included community Household heads and caregivers were interviewed about all children under 5 living in the household. All children aged 1–59 months were eligible for inclusion in the trial, and weight measurements for all children aged 1–11 months were collected as described below. Age was determined based on the date of birth in the child’s government-issued health card. All children aged 1–59 months were included in the census regardless of gestational age, but we did not collect information of gestational age or birthweight. Children under 3.8 kg were not eligible for treatment as part of the trial because of the concerns that small young infants may be at increased risk of infantile hypertrophic pyloric stenosis due to azithromycin,^16^^,^^22^ but weight measurements were collected for all children in the eligible age range and thus they were included in this analysis. Children who were sick at the time of the baseline census for the trial were not excluded from trial and thus are included in this analysis, but we did not collect information about morbidity at the time of the census.

### Anthropometric measurements.

Weight measurements were collected for infants aged 1–11 months to facilitate weight-based dosing with azithromycin or matching placebo (identical formulation to azithromycin with the exception of the active ingredient).^16^ A single weight measurement was taken for each infant using an electronic hanging infant scale (ADE M111600, Hamburg, Germany). The scale is calibrated by the manufacturer and has a precision of 20 g. Heavy clothes were removed from infants before weighing. The exact measurement from the electronic scale was entered. Weight measurements were collected by study team members performing the census who had been trained by study investigators on the infant weighing. Only a single weight measurement was collected because weight measurements were primarily used for calculation of treatment dosage and not for formal anthropometric assessment. Children aged 12–59 months were measured with a height stick to approximate weight-based dosing. As children aged 12–59 months did not have weight measurements available, we restricted the analytic population to children aged 1–11 months. Children over 6 months of age additionally had a single mid-upper arm circumference (MUAC) measurement taken using a standard 26-cm MUAC tape (Weigh and Measure, LLC, Olney, MD). We calculated WAZ scores using the 2006 WHO growth standards using the *zscore06* function in Stata 15.1 (StataCorp, College Station, TX). The WAZ values below −6 or above +5 were considered biologically implausible by the 2006 WHO growth standards and these observations were excluded from analyses.^23^ Children with WAZ < −2 were considered underweight, and those with WAZ < −3 were considered severely underweight. We used MUAC to classify children as moderate acutely malnourished (11.5–12.5 cm) or severe acutely malnourished (< 11.5 cm). Children with MUAC measurements < 11.5 cm were referred to a nutritional program.

### Geographic measurements.

During the baseline census for the trial, global positioning system (GPS) coordinates were collected for each household structure. The GPS coordinates were collected for each primary healthcare facility in Nouna District. Distance between the child’s household and their assigned primary healthcare facility was calculated using the *geodist* function in Stata,^24^ which calculates the geodetic distance between two points along a mathematical model of the earth’s surface.

### Socioeconomic status measurements.

Before the implementation of this study, all communities outside the Nouna HDSS were mapped and heads of households were interviewed about their household’s assets. Communities in the HDSS were not included in this exercise as the primary purpose was to map the location of each physical structure in each community to facilitate conduct of the parent trial. Communities and structures in the HDSS area have been previously mapped. In communities where household surveys were conducted, heads of households reported the type of latrine used by their household (improved, unimproved, or none/open defecation) and if the household owned a mobile phone or a radio. Improved latrines were defined as latrines with a slab and unimproved latrines those without slab.

### Statistical analysis.

Demographic characteristics were summarized by underweight status as defined by WAZ, using medians and interquartile ranges (IQR) for continuous variables and proportions for categorical variables. The WAZ was summarized using means and SDs. We evaluated the relationship between distance from the child’s household to their assigned primary healthcare facility and WAZ using linear regression models with Huber–White robust standard errors adjusted for clustering within communities.^25^ We first constructed a linear regression model in the entire study population adjusted for the child’s age and sex. We then fit the same model in the subpopulation (*N* = 177 communities) that had socioeconomic status measurements. Finally, we fit a model adjusted for the child’s age, sex, household latrine access, radio and mobile phone ownership to adjust for confounding by socioeconomic status. We then ran a series of logistic regression models evaluating the relationship between underweight (WAZ < −2) and distance to the healthcare facility, which were adjusted as described for the primary set of models evaluating WAZ. Potential sources of bias include confounding and selection bias given that socioeconomic status measurements were only collected in a subset of communities. We constructed a series of models that include both age- and sex-adjustment and socioeconomic status to minimize confounding. To evaluate potential differences in study samples between those with and without socioeconomic status data that could lead to selection bias, we compared descriptive characteristics, including medians and IQRs for continuous variables or means and SDs for WAZ and proportions for categorical variables. All statistical analyses were conducted in Stata 15.1 and figures were generated in R version 3.6.2 (The R Foundation for Statistical Computing).

## RESULTS

**Figure 1.**
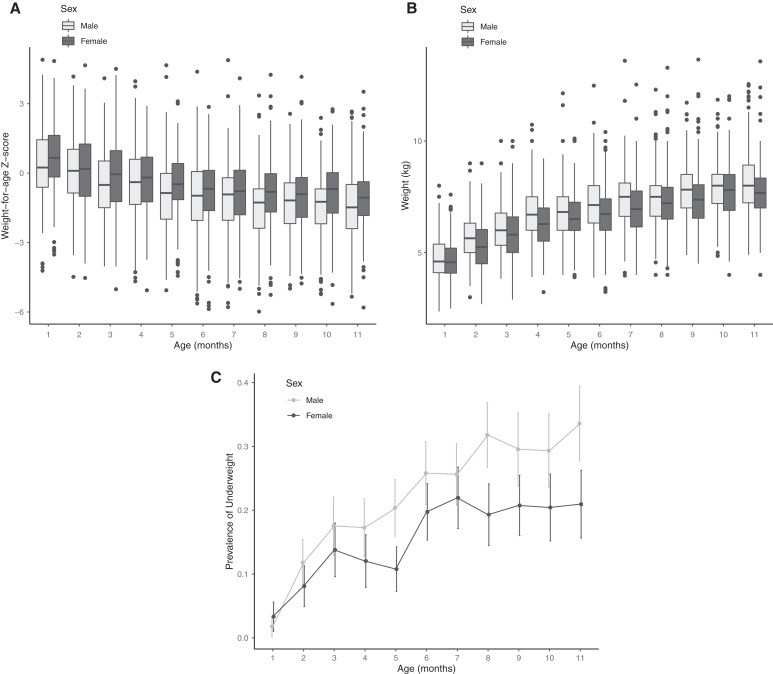
(**A**) Weight-for-age Z-score, (**B**) weight, and (**C**) prevalence of underweight (WAZ<−2, C) by child’s sex and age in months. Light gray indicates results for males and dark gray for females.

A total of 44,192 children aged 1–59 months were recorded in the census, of whom 7,109 (16.1%) were 1–11 months of age at the time of the census. Among these children, 6,465 (90.9%) had a weight measurement, of which 6,437 (99.9%) were considered valid based on WAZ. Of these, primary healthcare facility information and GPS coordinates were available for 6,077 (92.4%; 85.5% of children 1–11 months of age). Children in the analytic sample were a median of 6 months old (IQR 3 to 8) and 48.4% were female (Table 1). Mean WAZ was −0.68 (SD 1.6) and 1,152 were underweight (19.0%). The WAZ decreased with increasing age and boys had lower WAZ than girls (Figure 1A). Weight increased with age, and boys weighed more than girls (Figure 1B). The prevalence of underweight increased with increasing age from 2.5% among 1-month-olds to 27.6% among 11-month-olds. Disparities in underweight between boys and girls increased with increasing age (Figure 1C).

**Table 1:** Demographic characteristics of the study population (*N* = 6,077)

	Underweight* (*N* = 1,152)	Not Underweight (*N* = 4,925)
Age, months, median (IQR)	7 (5–9)	6 (3–8)
Child’s sex, *N* (%)		
Female	459 (39.8%)	2,481 (50.4%)
Male	693 (60.2%)	2,444 (49.6%)
Distance to clinic, km, median (IQR)	4.2 (1.2–6.5)	4.4 (1.4–6.8)
Mid-upper arm circumference,† cm, median (IQR)	13 (12.5–13.8)	14 (13.2–14.6)
Weight, kg, median (IQR)	5.7 (5.0–6.3)	7.1 (6.1–8.0)
Weight-for-age Z-score, mean (SD)	−2.96 (0.81)	−0.14 (1.19)
Household latrine type‡		
Improved	39 (4.4%)	208 (5.5%)
Unimproved	458 (52.0%)	2,038 (53.8%)
None	384 (43.6%)	1,543 (40.7%)
Household water source‡		
Borehole	117 (13.3%)	573 (15.1%)
Shallow dug well	764 (86.7%)	3,216 (84.9%)
Household mobile phone ownership,‡ *N* (%)	688 (78.5%)	3,065 (81.4%)
Household radio ownership,‡ *N* (%)	425 (48.9%)	1,850 (49.4%)

IQR = interquartile range.

* Defined as weight-for-age Z-score < -2.

† Measured only in children aged > 6 months.

‡ Measured in a subset of 177 communities with household socioeconomic status data.

**Figure 2.**
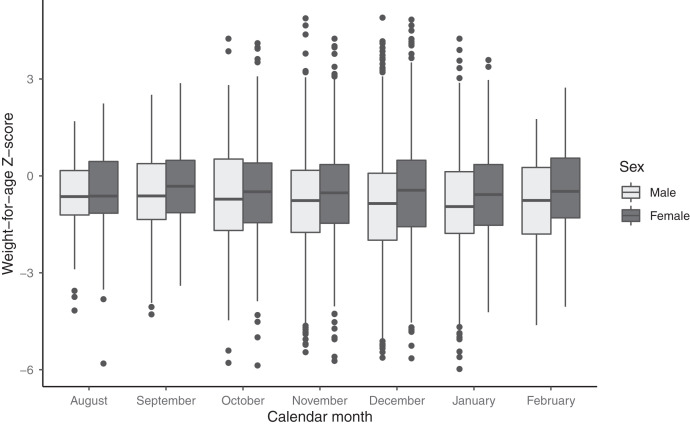
Weight-for-age Z-score by month of measurements. Light-shaded boxes indicate results for male and dark-shaded boxes for females.

Characteristics were generally similar between children with socioeconomic status data compared with those without (Supplemental Table 1), although median distance from the household to the primary healthcare facility was approximately 1 km longer among households with socioeconomic status data compared with those without. This is reflective of a higher density of healthcare facilities in the Nouna HDSS area compared with outside of the HDSS area (2.2 facilities per 1,000 children aged 1–59 months within the Nouna HDSS versus 0.8 facilities per 1,000 children outside of the HDSS). There were no differences in WAZ by calendar month of measurement (Figure 2).

Mid-upper arm circumference measurements were available for 2,675 (44.0%) of infants, all of whom were over 6 months of age. Median MUAC was lower among underweight compared with normal weight children (median 13 cm versus 14 cm, Table 1) and 262 children (9.8%) had moderate acute malnutrition and 26 (1.0%) had severe acute malnutrition based on MUAC. Mean WAZ was lower in children with moderate and severe acute malnutrition as determined by MUAC (Table 2, *P* < 0.001). The prevalence of moderate and severe acute malnutrition increased as WAZ decreased (Table 2).

**Table 2:** MUAC and underweight among children > 6 months (*N* = 2,675)

	*N*	WAZ Mean (SD)	WAZ ≥ 2 *N* (%)	WAZ < 2 to −3 *N* (%)	WAZ < −3 *N* (%)
Total			1,994	428	253
MUAC					
≥ 12.5 cm	2,413	−1.01 (1.3)	1,902 (95.4%)	346 (80.8%)	165 (65.2%)
< 12.5 to 11.5 cm	236	−2.29 (1.4)	89 (4.5%)	76 (17.8%)	71 (28.1%)
< 11.5 cm	26	−3.21 (1.0)	3 (0.2%)	6 (1.4%)	17 (6.7%)

MUAC = mid-upper arm circumference; WAZ = weight-for-age Z-scores.

**Figure 3.**
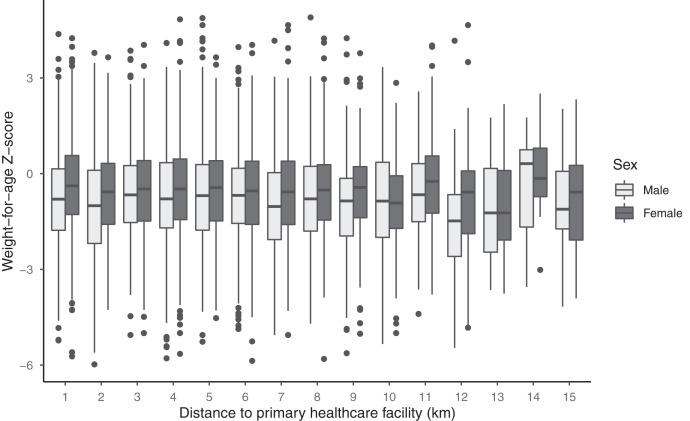
Weight-for-age Z-score by distance from the child’s household and the primary healthcare facility serving the child’s visit. Light-shaded boxes indicate results for male and dark-shaded boxes for females.

Median distance from the child’s household to the primary healthcare facility serving their community was 4.3 km (IQR 1.3–6.5), which was similar between children who were normal weight and underweight. Increasing distance from the primary healthcare facility was not associated with changes in WAZ (mean difference −0.01, 95% CI −0.03 to 0.006; Table 3 and Figure 3). This result did not change in models restricted to the population with socioeconomic status and adjusted for socioeconomic variables (Table 3) or for underweight versus non-underweight children (Supplemental Table 2). Improved latrine use by the household was associated with increased WAZ overall, and this effect was stronger in male compared with female infants (Table 4).

**Table 3:** Associations between weight-for-age Z-score and sociodemographic characteristics

	Age- and sex-adjusted only*	Age- and sex-adjusted only, SES sample*†	Age-, sex-, and SES-adjusted*†
	Mean difference (95% CI)	Mean difference (95% CI)	Mean difference (95% CI)
*N* in model	6,077	4,601	4,601
Age in months	−0.17 (−0.19 to −0.16)	−0.17 (−0.19 to −0.15)	−0.17 (−0.19 to −0.15)
Female sex	0.33 (0.25–0.41)	0.34 (0.25–0.43)	0.34 (0.25–0.43)
Distance to clinic, per kilometer	−0.01 (−0.03 to 0.006)	−0.02 (−0.04 to 0.007)	−0.01 (−0.04 to 0.008)
Latrine type			
Improved			Ref
Unimproved	N/A	N/A	−0.27 (−0.46 to −0.09)
None			−0.26 (−0.48 to −0.05)
Dug well use for water vs. borehole	N/A	N/A	−0.07 (−0.23 to 0.09)
Radio ownership	N/A	N/A	0.05 (−0.06 to 0.15)
Mobile ownership	N/A	N/A	0.10 (−0.02 to 0.22)

SES = socioeconomic status; N/A = not applicable.

* Linear regression model with standard errors adjusted for clustering within communities.

† Socioeconomic status was measured in a subgroup of communities (“SES sample”; *N* = 177) and these models are restricted only to the subpopulation with socioeconomic status measurements (*N* = 4,601).

**Table 4:** Sex-stratified associations between weight-for-age Z-scores and sociodemographic characteristics

	Males			Females		
	Age- and sex-adjusted only*	Age- and sex-adjusted only, SES sample*†	Age-, sex-, and SES-adjusted*†	Age- and sex-adjusted only*	Age- and sex-adjusted only, SES sample*†	Age-, sex-, and SES-adjusted*†
	Mean difference (95% CI)	Mean difference (95% CI)	Mean difference (95% CI)	Mean difference (95% CI)	Mean difference (95% CI)	Mean difference (95% CI)
*N* in model	3,137	2,401	2,401	2,940	2,200	2,200
Age, per month	−0.18 (−0.20 to −0.16)	−0.18 (−0.20 to −0.15)	−0.18 (−0.20 to −0.15)	−0.16 (−0.18 to −0.14)	−0.16 (−0.19 to −0.14)	−0.16 (−0.19 to −0.14)
Distance to clinic, per kilometer	−0.006 (−0.03 to 0.02)	−0.01 (−0.04 to 0.02)	−0.007 (−0.04 to 0.02)	−0.02 (−0.05 to 0.002)	−0.02 (−0.05 to 0.006)	−0.02 (−0.05 to 0.006)
Latrine type						
Improved			1.00			1.00
Unimproved	N/A	N/A	−0.37 (−0.65 to −0.09)	N/A	N/A	−0.17 (−0.44 to 0.10)
None			−0.30 (−0.60 to 0.008)			−0.24 (−0.52 to 0.05)
Dug well use for water vs. borehole	N/A	N/A	−0.14 (−0.36 to 0.07)	N/A	N/A	0.0009 (−0.20 to 0.20)
Radio ownership	N/A	N/A	0.01 (−0.13 to 0.16)	N/A	N/A	0.09 (−0.05 to 0.22)
Mobile ownership	N/A	N/A	0.19 (0.02 to 0.36)	N/A	N/A	0.01 (−0.15 to 0.17)

SES = socioeconomic status; N/A = not applicable.

* Linear regression model with standard errors adjusted for clustering within communities.

† In the subpopulation with socioeconomic status measurements (*N* = 4,601).

## DISCUSSION

Nearly one in five infants were underweight as defined by WAZ ≤ 2, with increasing prevalence of underweight with increasing age. This result is reflective of trends in the region, which bears a large burden of stunting, wasting, and underweight in preschool children.^26^ Undernutrition is declining in many areas of sub-Saharan Africa, including in Burkina Faso. In 1999, median WAZ was −1.8 and prevalence of underweight nearly 50% in children aged 6–30 months in the same study area, compared with WAZ −0.7 and prevalence of underweight of 19% in the current study.^27^^,^^28^ However, the two estimates are not directly comparable as the present study only includes data for children aged 1–11 months. In the present study, WAZ decreased and the prevalence of underweight increased with increasing age, similar to what has been reported in other settings.^29^ Exclusive breastfeeding is recommended in Burkina Faso for the first 6 months of a child’s life, although in practice the prevalence of exclusive breastfeeding is often low.^30^ Age-specific results indicate a rapid uptick in the prevalence of underweight at approximately 6 months of age, which may be related to exclusive breastfeeding until 6 months of age and then introduction of complementary foods that may provide inadequate nutrition.^31^ Children at this age may also be exposed to more enteric pathogens as they interact more with their environment or may be exposed to contaminated complementary foods, which may affect their nutritional status.^32^ However, we did not collect data on breastfeeding or dietary diversity in this study and thus are unable to comment on the prevalence of exclusive and complementary breastfeeding. At all ages, both WAZ and prevalence of underweight was higher among boys than girls, a difference that became more pronounced at older ages. Sex differences in nutritional status have been noted in other settings, including in early infancy and at later ages.^33^^–^^35^ Male infants in general have been shown to have worse outcomes compared with female infants, including increased incidence of and reduced probability of recovery from wasting.^36^ Specific reasons for these differences are unclear and likely context-specific, and may include both sociocultural norms related to feeding practices as well as underlying physiology that may make male infants more vulnerable to undernutrition.^34^

We evaluated several potential determinants of underweight, including seasonality, distance from the healthcare facility, and socioeconomic factors including access to improved sanitation. The WAZ and the prevalence of underweight did not substantially differ by distance to the healthcare facility in the present study. Children who are further from healthcare facilities may be less likely to receive antibiotics or other treatment of infections such as pneumonia or diarrhea that may reduce weight gain and promote undernutrition.^37^ Two possible explanations for this result include that there are no differences in care-seeking for underweight compared with normal weight children, or that increased distance from healthcare facilities does not influence healthcare utilization in the study area.^38^ There was no evidence of seasonal differences in WAZ in this analysis. Burkina Faso, as in much of the Sahel, experiences seasonal food insecurity during the rainy season (approximately July through October), with an annual harvest typically in November. Although we only collected data from August through February, this period covers the pre- and postharvest seasons, and we had hypothesized that WAZ would decrease until approximately December and then increase. At the population level, lack of evidence for seasonality in undernutrition may indicate that this age group is less vulnerable than others to fluctuations in food supplies. Improved latrine usage by the household was associated with significantly higher infant WAZ scores, particularly for male infants. Improved latrine usage may reduce exposure to enteric pathogens that can lead to diarrheal disease and adverse nutritional outcomes.^39^ Alternatively, latrine access may be a marker for overall socioeconomic status of the household that influences infant weight. Other household characteristics, including water source and socioeconomic status characteristics, were not associated with infant WAZ in this population.

The WAZ score has been suggested as a metric for predicting mortality in children under 5 years of age.^7^ Although MUAC is commonly use in community screening programs to identify children with acute malnutrition (wasting), its utility is less clear for children under 6 months of age. Previous work has indicated that WAZ reliably detects undernutrition in hospitalized infants under 6 months of age.^40^^,^^41^ In the present study, MUAC was not collected for children under 6 months of age as it was being used to screen for severe acute malnutrition in older children. However, in infants over 6 months of age, the prevalence of severe acute malnutrition was higher among children who were underweight and severely underweight, and mean WAZ was considerably lower among children with acute malnutrition compared with those without. The WAZ may be similarly useful as a screening tool for population-based samples of children under 6 months of age. Future studies evaluating the role of WAZ for predicting mortality among infants and its relationship with other anthropometric indices in children under 6 months of age will be useful for evaluating WAZ as a screening tool for targeting interventions in this population.

The results of this study must be considered in the context of several limitations. All weight measurements were collected on a hanging scale and were collected for weight-based dosing of children < 12 months who were participating in a randomized controlled trial of azithromycin compared with placebo for the prevention of child mortality.^16^ Children aged 12 months and older were measured with a height stick to approximate weight-based dosing (as is done in trachoma control programs), and thus did not have weight measurements and were not included in the present analysis.^16^ Duplicate measurements were not collected, and SD of WAZ was 1.6, indicating that there is likely some measurement error in weight measurements.^42^ We did not have longitudinal anthropometric or mortality data and are unable to comment on whether early-life underweight affects the growth failure or mortality. Understanding longitudinal growth trajectories in children and how early-life underweight status affects these trajectories will be important. Weight measurements were not collected in children aged 12 months and older, because they were weighed with a height-stick to approximate weight-based dosing, as is done in trachoma control programs.^43^^,^^44^ Although this approach to dosing facilitates mass drug administration, as a result we do not have weight measurements for older children and are unable to comment on undernutrition in children aged 12–59 months with these data.

These data arose from a large simple trial that was not designed specifically to evaluate determinants of underweight. As a result, we did not collect maternal or dietary data for children, which may have been useful for contextualizing results and understanding drivers of undernutrition. Important determinants of early undernutrition likely include maternal factors such as *in utero* exposures such as prenatal nutritional supplementation, which we were unable to evaluate in this study.^36^ Data on sociodemographic characteristics for household members other than children were not available, and we were unable to conduct structured observations for household sanitation facilities. Finally, our estimated distance would not necessarily reflect the amount of time taken to travel to a health clinic. Although the terrain is fairly flat in this area, road quality and availability vary widely, which can affect travel times. We did not collect data from households on travel time or travel modalities to healthcare facilities.

In summary, we documented a high prevalence of underweight that increased with age and was higher among boys than girls in a population-based sample of infants in rural Burkina Faso. These results add to a growing evidence base that early-life undernutrition is common and underscores the importance of development of nutritional interventions that can be implemented in young infants. Further research evaluating longitudinal trends in growth and mortality in underweight infants will be useful for evaluating the utility of WAZ as a screening tool for at-risk infants in similar settings.

## Supplemental tables


Supplemental materials
