# Supplementary material for: Epidemiology of Underweight among Infants in Rural Burkina Faso
Source: Am J Trop Med Hyg. 2021 Oct 25;106(1):361–8. doi: 10.4269/ajtmh.21-0838 (PMC8733516; doi:10.4269/ajtmh.21-0838)
Supplement: Supplementary file 1 [file tpmd210838.SD1.pdf]

Supplementary Table 1. Demographic characteristics among those with and without SES data

	With SES Data (N=4,601)	Without SES Data (N=1,476)
Number of communities	177	52
Age, months, median (IQR)	6 (3 to 9)	6 (3 to 8)
Female sex, N (%)	2,200 (47.8%)	740 (50.1%)
Distance to clinic, km, median (IQR)	4.5 (1.7 to 6.7)	3.4 (0.9 to 6.1)
Weight, kg, median (IQR)	6.9 (5.8 to 7.8)	6.6 (5.6 to 7.5)
Weight-for-age Z-score, mean (SD)	-0.68 (1.60)	-0.70 (1.55)
Underweight, N (%)	867 (18.8%)	285 (19.3%)
Mid-upper-arm circumference, cm, median (IQR)[1]	13.8 (13 to 14.5)	13.5 (13 to 14.5)

Abbreviations: SES, socioeconomic status; IQR, interquartile range; km, kilometer; kg, kilogram; SD, standard deviation; cm, centimeter; [1]Among children >6 months of age

Supplementary Table 2. Associations between underweight (weight-for-age Z-score<-2) and distance to clinic

	Age- and sex-adjusted only[1]	Age- and sex-adjusted only, SES sample[1,2]	SES-adjusted[1,2]
	Odds Ratio (95% CI)	*Odds Ratio (95% CI)*	*Odds Ratio (95% CI)*
N	6,077	4,601	4,601
Distance to clinic, per km	1.02 (0.99 to 1.05)	1.02 (0.99 to 1.05)	1.02 (0.98 to 1.05)
Age in months	1.15 (1.13 to 1.20)	1.16 (1.12 to 1.20)	1.16 (0.12 to 1.20)
Female sex	0.64 (0.56 to 0.72)	0.65 (0.56 to 0.75)	0.65 (0.56 to 0.75)
Latrine type<br>Improved<br>Unimproved<br>None	N/A	N/A	 1.00<br>1.30 (0.83 to 2.04)<br>1.37 (0.87 to 2.17)
Radio ownership	N/A	N/A	0.97 (0.81 to 1.15)
Mobile ownership	N/A	N/A	0.90 (0.73 to 1.11)

Abbreviations: SES, socioeconomic status; CI, confidence interval; N/A, not applicable; [1]Logistic regression model with standard errors adjusted for clustering within communities; [2]In the subpopulation with socioeconomic status measurements (N=4,061)
